# Identification of an additional deep intronic splice variant prompts critical evaluation of *SPG7* inheritance

**DOI:** 10.1007/s10048-026-00882-7

**Published:** 2026-02-09

**Authors:** Emma H Gillesse, Miranda Wan, Setareh Ashtiani, Oksana Suchowersky, Jillian S Parboosingh, Francois P Bernier, Ryan E Lamont, A Micheil Innes, PY Billie Au, Care4Rare Canada Consortium

**Affiliations:** 1 Alberta Children’s Hospital Research Institute, Calgary, Canada; 2 Department of Medical Genetics, University of Calgary, Calgary, Canada; 3 Department of Clinical Neurosciences, University of Calgary, Calgary, Canada; 4 Departments of Medicine (Neurology) and Medical Genetics, University of Alberta, Edmonton, Alberta, Canada

**Keywords:** Hereditary spastic paraplegia, Genome sequencing, Missing heritability, SPG7, Spastic paraplegia 7, Autosomal dominant, Autosomal recessive

## Abstract

*SPG7*-related hereditary spastic paraplegia (*SPG7*-HSP) is one of the most common forms of autosomal recessive HSP. There is a growing number of reports of affected individuals found to be heterozygous carriers for the recurrent pathogenic coding variants in *SPG7*, most notably p.Ala510Val, and this has further led to the suggestion of *SPG7*-HSP having both recessive and dominant forms. Here, we report a proband with pure HSP initially found to carry a heterozygous pathogenic stop-gain variant in SPG7 (NM_ 003119.4:c.1672 A > T; p.Lys558Ter). Subsequent short-read genome sequencing (GS) identified a second, novel deep intronic variant (NM_003119.4:c.987 + 152G > A) in trans, predicted to activate a cryptic splice donor site. RNA sequencing confirmed inclusion of intronic sequence, resulting in a frameshift and premature stop codon (p.Ser330ValfsTer10). This is only the second report of GS uncovering a pathogenic deep intronic variant in *SPG7.* Our findings highlight that variants only detectable by GS may be an underappreciated disease mechanism and may account for the missing heritability in instances where only a single coding variant is initially identified. Further, critical review of such reports in the literature found no substantial evidence for true autosomal dominant inheritance in *SPG7-HSP*. These cases most likely represent undetected second variants, alternative molecular diagnoses, or more complex disease mechanisms that have yet to be understood. We recommend the use of GS in individuals with suspected SPG7-HSP carrying only a single pathogenic variant to ensure a complete and accurate molecular diagnosis.

## Introduction

Hereditary spastic paraplegia (HSP) is a group of rare, phenotypically and genetically heterogeneous neurodegenerative disorders. Currently, over 80 genes have been associated with HSP with autosomal dominant (AD), autosomal recessive (AR), X-linked, and mitochondrial patterns of inheritance [1]. AD- and AR-HSP are the most common, both respectively predicted to have a prevalence of 1.8 per 100,000 individuals [2]. HSP can be described as ‘pure’, presenting with progressive lower-extremity spasticity and weakness, diminished proprioception, and variable hypertonic urinary bladder disturbances. HSP is considered ‘complex’ when an individual has additional features such as ataxia, intellectual disability, seizures, muscle atrophy, and optic atrophy. Further, there can be significant phenotypic overlap with other disorders such as amyotrophic lateral sclerosis (ALS), primary lateral sclerosis, hereditary neuropathies, spastic ataxias, and hereditary cerebellar ataxia. This phenotypic variability and non-specificity represent one of the largest challenges in clinically diagnosing individuals with HSP, and confirmatory molecular testing is often necessary [3].

*SPG7*-related HSP (*SPG7*-HSP) is one of the most common forms of AR-HSP, accounting for an estimated 4.5–12.5% of HSP cases, and has a global prevalence of 0.22 per 100,000 people [4–6]. Onset can vary from infancy to adulthood in different families. *SPG7* encodes the inner mitochondrial membrane protease, paraplegin, which plays key a role in quality control of proteins being imported into the mitochondria, including the degradation of misfolded proteins. *SPG7* was the first HSP locus to be mapped [7] and has been associated with both pure and complex AR-HSP. More recently, individual case reports have linked the gene to a variety of other disorders including Parkinson’s disease [8], ALS [9], progressive muscular atrophy [10], and isolated dominant optic atrophy [11–13].

There are a growing number of reports suggesting *SPG7* can lead to autosomal dominant HSP (AD-HSP) as well. Although uncommon, this phenomenon of genes being associated with both recessive and dominantly inherited disorders has been observed before. Examples include *ALDH18A1* [14, 15] and *REEP2* [16, 17] which are genes that are associated with both AR-HSP and AD-HSP. However, the validity of autosomal dominant *SPG7*-HSP remains contentious given the limited reports of multi-generational families or cases with de novo inheritance.

The majority of *SPG7*-HSP studies have used either Sanger-sequencing or capture-based panel or exome sequencing. This approach is typically successful and has identified several reoccurring pathogenic single nucleotide variants (SNVs), including the well known p.Ala510Val variant. This variant is by far the most commonly reported variant in the gene, with reports finding up to 58.5–65% of individuals with *SPG7*-HSP to be either compound heterozygous or homozygous for the variant [11, 18]. Several studies have additionally used multiplex ligation-dependent probe amplification (MLPA) to detect exonic copy number variants (CNVs) in *SPG7*, and a range of multi-exon deletions have been reported in the literature [18–22].

There is currently only one case report reporting use of short-read genome sequencing (GS); Verdura et al. [23] used GS to identify a pathogenic deep intronic splice variant in trans with a previously identified pathogenic coding *SPG7* SNV, in an individual with AR-HSP. Beyond this single report, little is known about non-coding variants in *SPG7*.

We report an individual with pure HSP who was identified by commercial panel sequencing to have a single heterozygous pathogenic stop-gain variant in *SPG7*. GS was pursued to identify as second variant in the gene, and a deep intronic splice variant was detected. Subsequent RNAseq analysis revealed the variant to result in inclusion of intronic sequence and ultimately a premature stop codon. This report adds to emerging evidence that non-coding variants detectable only by GS may represent an important overlooked disease mechanism in *SPG7*-HSP. We further discuss the implications of these potentially overlooked variants in our current understanding of *SPG7*-HSP inheritance.

## Clinical report

The adult female proband was first referred to Medical Genetics at 62-years of age for spastic paraplegia. She reported that her symptoms started at age 45 with increasing lower extremity stiffness and occasional falls due to tripping and dragging her feet. She was athletic as a child and young adult with no early motor concerns.

Her family is of English ancestry with no consanguinity. Her sister also has a similar gait disorder with hyperreflexia and spasticity in her legs which started in her 50s. Unfortunately, no further information was available regarding clinical investigations for this sister.

Her brother passed away from stroke. Her father had an unspecified dementia. Her mother had a history of emphysema and it is unclear if she had mobility difficulties due to this or an additional gait disorder. Her maternal grandfather had Parkinson’s disease. She has three children and three grandchildren who are currently healthy.

On exam documented at age 49 years, she had brisk reflexes in the upper extremities with slight spread, positive Hoffmann’s reflex, and markedly brisk reflexes with spread in her lower extremities. She had increased tone in the lower extremities. Plantar reflexes were upgoing bilaterally. Her gait showed a mild, spastic diplegic pattern. MR brain, C, T, and L spine completed did not reveal a cause for her symptoms.

Over the next 10 years, she had slow progression of her symptoms, with inability to continue working and unable to manage stairs. She used ankle-foot orthoses. She trialed baclofen and tizanidine for spasticity but had intolerable side effects and no benefit.

By her mid-50s, she had progressed to needing two-wheeled walker due to recurrent falls. In her early 60s, she was treated with lower extremity botulinum toxin injections which improved her gait pattern. She developed bladder urgency and frequency and was diagnosed with neurogenic detrusor overactivity in her early 50swhich was also treated with botulinum injections with improvement in her symptoms.

By her mid 60s, her neurologic examination was significant for an increased jaw jerk. Upper extremities remained relatively unaffected aside from brisk reflexes but lower extremities demonstrated increased tone and hyperreflexia with spread, mild hip flexor weakness and moderate foot dorsiflexor weakness. Plantar reflexes revealed bilateral upgoing toes. Sensory testing for pinprick, temperature, vibration, and proprioception were normal. Her diagnosis was felt to be most in keeping with hereditary spastic paraplegia.

She was assessed through Medical Genetics and offered a 230-gene combined Spastic Paraplegia and Ataxia Panel performed commercially through Blueprint Genetics. This identified a heterozygous known pathogenic *SPG7* variant NM_ 003119.4: c.1672 A > T, p.(Lys558Ter). This ClinVar pathogenic variant (VCV000188276.34) has an allele count of 239 heterozygotes and no homozygotes in Gnomad v4.1.0 [accessed Sept. 9, 2025], and has been reported in both homozygous and compound heterozygous affected individuals [20, 24, 25]. No additional variants were identified in *SPG7*.

## Results

### Genetic testing

Analysis of GS data identified the previously reported NM_ 003119.4 (*SPG7*): c.1672 A > T p.(Lys558Ter) variant, as well as a deep intronic variant in intron 7, NM_003119.4 (*SPG7*):c.987 + 152G > A. This variant is not currently present in GnomAD v4.1.0 [accessed Sept. 9, 2025] and has not been reported in the literature. SpliceAI [26] predicted this to result in the activation of a nearby cryptic splice donor site (Δ score: 0.64). The phase of the c.987 + 152G > A and c.1672 A > T variants was determined via Sanger sequencing of genomic DNA from the proband’s two daughters. Both daughters were found to carry only the c.987 + 152G > A intronic variant, confirming the variants to be in trans in the proband.

**Fig. 1:**
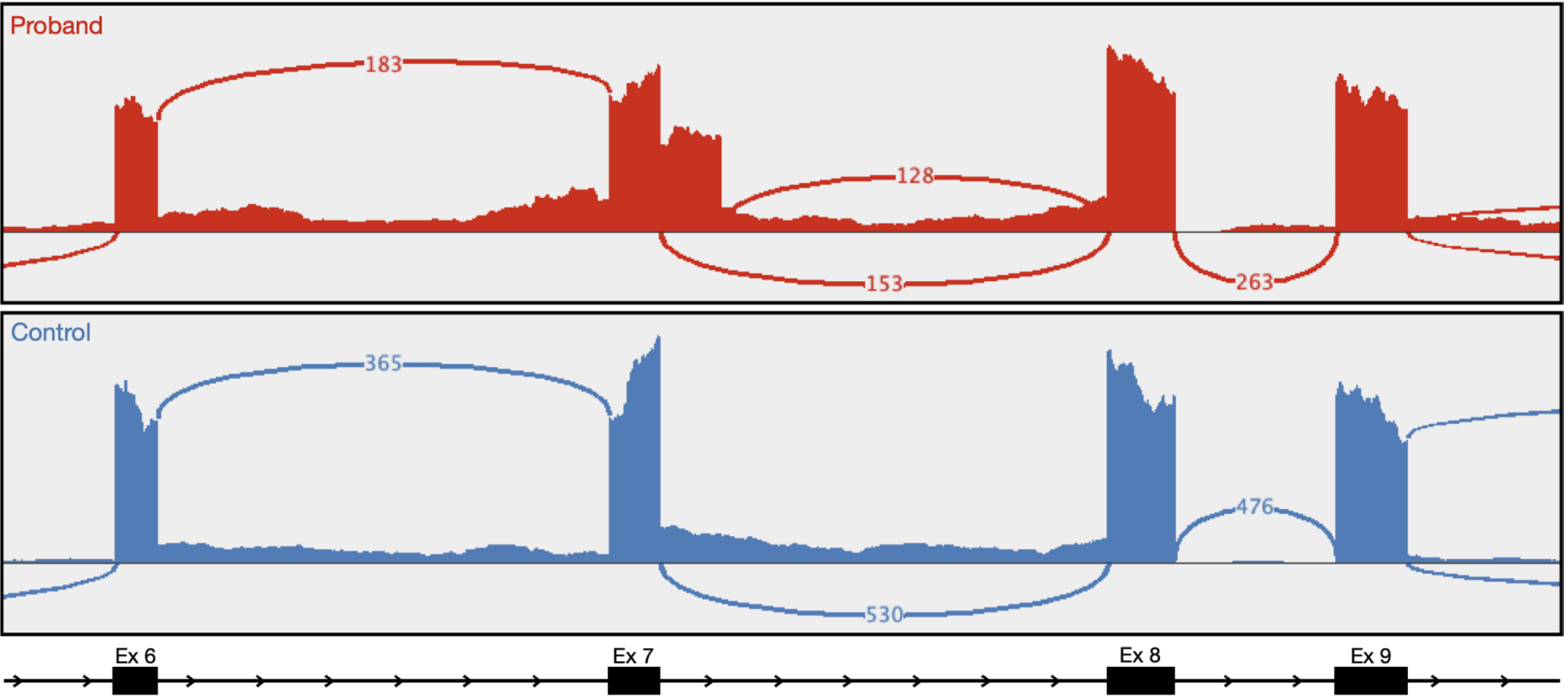
IGV generated sashimi plot from RNAseq data from blood for our proband (top) and an age and sex matched control (bottom). Sequencing and junction coverage for our proband shows inclusion of intronic sequence in exon 7 as a result of NM_ 003119.4 (*SPG7*):c.987 + 152G > A. Splice junctions labeled with the number of supporting reads. Exons labeled corresponding to NM_ 003119.4 transcript

RNAseq analysis revealed that the c.987 + 152G > A variant resulted in the creation of an intronic cryptic splice donor site that leads to inclusion of the first 149 base-pairs of intron 7 in the mRNA, ultimately leading to a frameshift and premature stop gain; r.987_988ins149 p.(Ser330ValfsTer10) (Fig. 1). This novel splice junction was not detected in an age, sex, and tissue matched control (Fig. 1). This was orthogonally validated using cDNA from blood (RT-PCR and then Sanger sequencing) from the proband’s daughter who has the c.987 + 152G > A variant (data not shown).

## Discussion

Here we present an individual with pure HSP, for whom a complete molecular diagnosis would not have been achieved without the use of GS. This analysis approach identified compound heterozygous variants in *SPG7*: a previously reported pathogenic variant, c.1672 A > T (p.Lys558Ter), and a novel deep intronic splice variant, c.987 + 152G > A (p.Ser330ValfsTer10). After Verdura et al. [23], this is the second report of GS being used with the intent of identifying a deep intronic pathogenic variant to confirm a diagnosis of *SPG7*-HSP.

This report highlights a key benefit of using GS over other commonly reported approaches for *SPG7*-HSP genetic testing: GS offers detection of non-coding and structural variants in addition to coding SNVs. We further discuss how past inability to interrogate non-coding regions of the genome and limited access to GS has impacted the current understanding of *SPG7*-HSP inheritance. *SPG7* is currently listed in OMIM as having AD- and AR-HSP. This is in part due to several reports of affected individuals having only a single coding variant in *SPG7* [11, 18, 19, 21, 27–31]. However, none of these reports of observed AD *SPG7*-HSP demonstrate a single, appropriately rare heterozygous variant either segregating in multiple affected individuals from multiple generations of a family, or confirmed de novo inheritance in an affected individual with genetically confirmed unaffected parents. Further, these reports are directly confounded by the limitations of the genetic testing methods used, and there are several likely alternative explanations for these observations that could be addressed with GS.

The first report to suggest the possibility of *SPG7*-HSP came from McDermott et al. in 2001 [27]. Among a cohort of families with HSP, one affected individual was found to have inherited the common p.Ala510Val variant from his mother, and the variant p.Arg485_Glu487del from his father, consistent with autosomal recessive inheritance. Upon further clinical evaluation, the proband’s father was also felt to have a milder phenotype consistent with HSP. No other variants were identified in *SPG7* for the father by simultaneous single-stranded conformation analysis and heteroduplex analysis, and Sanger sequencing, which lead the authors to suggest he had a dominant form of *SPG7*-HSP.

Since this first report, there have been several reports of dominant inheritance following this same pattern of a proband with two compound heterozygous variants, and an ostensibly affected parent or child with one heterozygous variant [11, 18, 19, 28]. As it has become more accepted that *SPG7*-HSP can have both recessive and dominant inheritance, several reports have subsequently labelled individuals who have a single heterozygous *SPG7* variant, as having a dominant form of HSP [21, 28–31]. None of these reports segregated the variants in parents to confirm inheritance, and many did not formally clinically evaluate family members described to be affected.

These reports commonly suggest the same three variants to be dominant acting: p.Leu78Ter, p.Arg485_Glu487del, and p.Ala510Val. These are among the most commonly reported variants in instances of confirmed AR-*SPG7*-HSP, and no plausible mechanism for how they are also dominant acting has yet to be proposed. Furthermore, it is highly unlikely that these variants can be dominant acting based on their observed allele frequency. As *SPG7*-HSP is a rare disease, autosomal dominant pathogenic variants should be appropriately rare and relative to the frequency of the disease in the general population. AD-HSP has an estimated global prevalence of 1.8 per 100,000 individuals (0.0018%) [2], and *SPG7*-HSP specifically is estimated to affect 0.22 per 100,000 individuals (0.00022%) [6]. The p.Leu78Ter, p.Arg485_Glu487del, and p.Ala510Val variants have allele frequencies of 0.027%, 0.035%, and 0.579%, respectively (GnomAD v4.1.0 [accessed Sept. 9, 2025]).

It is understood that pathogenic variants associated with autosomal dominant adult-onset disorders may be present in healthy population databases due to individuals being included in these sequencing studies before the onset of disease. It has been determined that over 75% of ClinVar pathogenic variants associated with autosomal dominant adult-onset disease are not reported in the healthy population, and further only 5% of variants have a frequency over 0.01% [32]. Based on age specific data available in GnomAD v4.1.0, the allele frequencies for the p.Leu78Ter, p.Arg485_Glu487del, and p.Ala510Val variants in individuals over the average age of onset for *SPG7-HSP* (35-41.7 years) [18–20, 31, 33], are 0.037%, 0.076%, and 1.42%, respectively. Although variable penetrance may also contribute to pathogenic dominant variants being present in the healthy population, this explanation does not completely reconcile the discrepancy between the observed allele frequency for these variants and the estimated global prevalence of *SPG7*-HSP or AD-HSP more broadly [2, 6].

There are several likely alternative explanations for the observations made in these reports that should be further addressed before considering the possibility of AD-*SPG7*-HSP. First, most of reports suggesting autosomal dominant inheritance only sequenced *SPG7* or a small handful of HSP genes, and use of ES has been relatively limited in the prior literature. Cases that use a restricted hypothesis driven approach would not be able to exclude an alternative molecular diagnosis in one of the approximately 80 HSP genes recognized to date or in a novel HSP gene, including genes with established autosomal dominant inheritance. Additionally, there are several other genetic and acquired neurological disorders that can have significant phenotypic overlap with HSP, such as genetic and non-genetic forms of ALS and other motor neuron disease, Parkinson’s disease, neuropathy, and cerebellar ataxia [1, 3]. It is possible the disease-causing variant or variants lay in an entirely different subset of genes or is attributed to an alternative cause.

It is also possible that individuals do have *SPG7*-HSP, and their second pathogenic variant can only be detected with more advanced sequencing technologies, like GS. Although sequencing of the coding regions of *SPG7* and supporting MLPA has been successful in many cases, as previously mentioned, there are several types of potentially pathogenic variants that cannot be detected using this approach. As demonstrated by this report and that of Verdura et al. [23], ES cannot detect deep intronic variants that may impact splicing. GS also offers detection of more complex structural variants beyond CNVs, as well as coding and non-coding repeat expansions [34, 35].

An undetected second variant could be resulting in pseudodominant inheritance, which could explain the several reports of an affected individual with only a single heterozygous variant but their affected parent or child is compound heterozygous. Although uncommon, pseudodominance has been observed in *SPG7*-HSP before. van Gassen et al. [20] reported an affected father and son, one homozygous for the p.Ala510Val variant, and the other compound heterozygous for that variant and the p.Gly349Ser variant.

It is also a possibility that these observed heterozygous *SPG7* variants contribute to more complex genetic disease mechanism, such as digenic or polygenic inheritance. The possibility of non-Mendelian inheritance in SPG7-HSP has been proposed, including digenic inheritance with *AFG3L2* [36, 37], however further evidence is need to support this possibility.

The importance of properly understanding the inheritance patterns of monogenic rare diseases cannot be understated. There can be significant implications when incorrect assumptions are made. First, inheritance patterns are essential for understanding disease risk for future children, or carrier status in other family members. This could impact an individual’s decision whether to pursue having children, or for family members to undergo genetic testing themselves. In the case of *SPG7*, an assumption of autosomal dominant inheritance would indicate a potential 50% recurrence risk, which could significantly impact an individual’s decision making. As we also discussed, assumption of an incorrect inheritance pattern could lead to the correct molecular diagnosis being overlooked. Not only does this render any future genetic testing uninformative, but it may also mean that the affected individual receives improper treatment or is counselled incorrectly on the progression of their disease.

We reiterate that there is currently no substantial evidence demonstrating true autosomal-dominant inheritance of *SPG7*-HSP. Further, our report and that of Verdura et al. [23] suggest that deep intronic splice variants may be an underappreciated disease mechanism in *SPG7*. We recommend that affected individuals who are only found to have a single heterozygous pathogenic variant in *SPG7* receive GS to look for deep intronic splice variants or structural variant on the other allele. Additionally, genetic analysis should be expanded to include other HSP genes and genes with known phenotypic overlap.

## Methods

### Consent to participate

The proband was enrolled in Care4Rare-SOLVE and recruited from the Alberta Children’s Hospital Medical Genetics Clinic. The study was approved by the Calgary Conjoint Health Research Ethics Board and informed written consent was obtained for all participants.

### Short-read genome sequencing (GS)

The proband received GS performed at The Centre for Applied Genomics in Toronto, Canada, using an Illumina Novaseq 6000 platform with at least 30X coverage across 90.5% of the genome and an average coverage of 54X. Bioinformatic processing and variant calling was performed as previously described for other Care4Rare and Care4Rare-SOLVE projects [38, 39]. Rare variants in *SPG7* were prioritized first. Non-coding variants were assessed based on predicted impact on splicing using SpliceAI [26].

### Phasing and splice analysis of NM_003119.4 (*SPG7)* :c.987 + 152G > A

*SPG7* variant phasing was performed by Sanger sequencing of genomic DNA from the proband’s two daughters. DNA was extracted from blood by Alberta Precision Laboratories using Gentra Puregene Blood Kits (Qiagen). Sanger sequencing was performed using BigDye Terminator v3.1 Cycle Sequencing Kit (Applied Biosystems) and visualized using capillary electrophoresis on a Hitachi 3130 genetic analyzer (Applied Biosystems).

### RNAseq

Whole-transcriptome RNAseq from whole-blood was used to determine the splice impact of the NM_003119.4 (*SPG7*):c.987 + 152G > A variant. Blood was collected from the proband in PAXgene Blood RNA tubes (BD Biosciences) and the RNeasy Plus Mini Kit (Qiagen) was used for RNA extraction. Library preparation was performed using the NEBNext Ultra II Directional RNA Library Prep Kit for Illumina. Sequencing was then performed at The Centre for Applied Genomics (Toronto, Canada) on an Illumina NovaSeq 6000 using an S4 flow cell. Bioinformatic processing was performed as described by previous Care4Rare Canada publications [40]. RNAseq data from an age and sex matched control individual with no rare damaging variants in *SPG7* was generated following the same protocol.

## Data Availability

The data that support the findings of this study are available upon request from the corresponding author.
